# Effect of deep brain stimulation on dysphagia in Parkinson’s disease: mechanisms, evidence, and outlook

**DOI:** 10.3389/fnagi.2025.1734432

**Published:** 2026-01-15

**Authors:** Jule Hofacker, Bahne H. Bahners, Cinja Huber, Christian J. Hartmann, Inga Claus, Sonja Suntrup-Krueger, Alfons Schnitzler, Tobias Warnecke, Bendix Labeit

**Affiliations:** 1 Department of Neurology, Medical Faculty and University Hospital Duesseldorf, Heinrich-Heine University Duesseldorf, Duesseldorf, Germany; 2 Medical Faculty and University Hospital Duesseldorf, Institute of Clinical Neuroscience and Medical Psychology, Heinrich-Heine University Duesseldorf, Duesseldorf, Germany; 3 Department of Neurology, University Hospital Muenster, Muenster, Germany; 4 Department of Neurology and Neurorehabilitation, Klinikum Osnabrueck – Academic Teaching Hospital of the WWU, Muenster, Germany

**Keywords:** deep brain stimulation, dysphagia, Parkinson’s disease, swallowing function, narrative review

## Abstract

**Introduction:**

Oropharyngeal dysphagia (OD) is a common and significant complication of Parkinson’s disease (PD), contributing to malnutrition, respiratory complications and impaired medication intake. The pathophysiology of OD in PD is heterogeneous, involving basal ganglia dysfunction with associated motor impairments in the oropharynx, cortical pathophysiology, and *α*-synuclein pathology in peripheral nerves. While deep brain stimulation (DBS) is an established intervention for motor symptom management in PD, its effects on swallowing function remain poorly understood and controversial. This narrative review aims to critically evaluate the current evidence on the effects of DBS on OD in PD and to outline potential future research directions, grounded in current understanding of OD pathophysiology and DBS mechanisms.

**Methods:**

A narrative review of clinical studies examining the effects of DBS on swallowing function in people with PD was conducted. Studies were identified through database searching of MEDLINE, Embase and Cochrane Library, from inception of the databases until May 2025. Inclusion criteria encompassed clinical studies and case reports investigating DBS effects on swallowing outcomes in people with PD, with no language restrictions applied. Data regarding study design, DBS intervention and stimulation parameters, swallow-related outcomes and assessment methods were extracted and compiled systematically.

**Results:**

A total of 24 clinical studies, including prospective and retrospective observational studies and case reports, were included in this review. Evidence regarding DBS effects remains inconsistent. Subthalamic DBS shows the greatest variability: some studies report improvements in aspiration frequency or pharyngeal timing, while others describe no change or even long-term deterioration in swallowing safety. Pallidal DBS appears to neither improve nor deteriorate swallowing function, however, evidence is limited to four mainly retrospective studies with small sample sizes. Evidence on combined or alternative targets remains sparse and heterogeneous. Patient-reported swallowing outcomes are often more favorable than instrumental measures.

**Discussion:**

DBS may influence swallowing in PD, but outcomes likely depend on OD phenotypes, stimulation targets and parameters. Future research should recruit adequately powered cohorts, apply standardized instrumental assessments including detailed OD phenotyping, systematically explore stimulation parameters, distinguish short- from long-term effects, and integrate OD outcomes into DBS programming.

## 1 Introduction

Oropharyngeal dysphagia (OD) is a common and clinically significant complication in people with Parkinson’s disease (PD), with prevalence rates reaching 80% when the gold-standard assessments, Flexible Endoscopic Evaluation of Swallowing (FEES) and Videofluoroscopic Swallow Study (VFSS), are used (Gong et al., 2022; Takizawa et al., 2016). Although commonly associated with advanced stages of PD, OD can also manifest as an early symptom (Claus et al., 2020; Pflug et al., 2018). It poses serious health risks, including malnutrition (Gültekin et al., 2024), aspiration pneumonia, and other respiratory complications (Won et al., 2020), increased mortality, secondary morbidity (Di Luca et al., 2021) and hospitalizations (Dilmaghani et al., 2022). Moreover, it can impair oral medication intake, potentially exacerbating motor fluctuations (Labeit et al., 2022b). Reliable assessment and treatment of OD is therefore essential.

Patients with PD often present with reduced awareness of their swallowing difficulty (Buhmann et al., 2019; Rangwala et al., 2025; Monteiro et al., 2014; Kalf et al., 2012), and silent aspiration without overt protective reflexes is common (Martell et al., 2024), which complicates detection of OD. Therefore, instrumental evaluations are essential for accurately detecting OD. Both FEES and VFSS are considered diagnostic gold standards, providing robust and reliable information on OD pathologies (Labeit et al., 2022a). However, FEES is particularly well-suited for patients with PD, as symptom fluctuations may require repeated assessments without the risk of radiation exposure. Additionally, it enables characterization of the OD phenotype. Phenotyping of OD categorizes swallowing disorders based on characteristic instrumental patterns rather than severity alone (Warnecke et al., 2021). Neurogenic OD represents a multietiologic syndrome with different phenotypic patterns depending on the underlying disease (Warnecke et al., 2021).

Although clinically relevant, OD is rarely the primary focus when selecting and implementing treatments such as deep brain stimulation (DBS). DBS is an established advanced therapy for patients with PD and medication-refractory tremor or motor fluctuations, and has become standard of care with substantial advances in technology and clinical management in recent years (Krauss et al., 2021). DBS involves the surgical implantation of electrodes into deep brain structures, commonly the subthalamic nucleus (STN) or the globus pallidus internus (GPi). These electrodes deliver continuous electrical stimulation with adjustable parameters including voltage, pulse width and frequency, to optimize therapeutic efficacy and minimize side effects. DBS effectively alleviates core motor symptoms such as tremor, rigidity, and bradykinesia, and often reduces dopaminergic medication needs (Hartmann et al., 2019).

Current evidence on DBS effects on swallowing function remains limited and partly contradictory. While some studies suggest improvements (Ciucci et al., 2008), others find no significant effect (Smith-Hublou et al., 2024) or report deterioration (Troche et al., 2014) in specific aspects of swallowing. These inconsistencies may stem from methodological differences, diverse patient populations, variations in stimulation targets and parameters, but also from the multifaceted nature of OD, which differs from other PD symptoms. Previous reviews on this topic have examined the effects of DBS on swallowing in PD in recent years. However, these publications largely provide descriptive summaries of study outcomes and do not integrate current advances in our understanding of OD pathophysiology, DBS mechanisms, stimulation parameter effects, or phenotype-specific swallowing patterns. The present review aims to address these gaps by offering a pathophysiology-based and mechanism-oriented synthesis of the evidence. In addition, we incorporate newly published clinical studies and systematically evaluate stimulation settings, swallowing protocol quality, and methodological limitations that critically shape interpretation of DBS effects. This approach provides a refined perspective on why swallowing outcomes remain heterogeneous and outlines how future studies can be designed to overcome the limitations of the existing evidence base.

### 1.1 Pathophysiology of OD in PD

The pathophysiology underlying OD in PD is not yet fully understood and is presumed to be heterogeneous, involving both central and peripheral mechanisms.

Among the central mechanisms, the dopaminergic basal ganglia system plays a pivotal role being both a primary site affected by neurodegeneration in PD and a critical component of the supramedullary motor control of swallowing (Wei et al., 2024). Clinically, it is therefore unsurprising that classical motor symptoms of PD, such as rigidity and bradykinesia, may also affect the oropharynx and contribute to OD (Labeit et al., 2020b). For example, analogous to freezing of gait, dysfunction in motor initiation and sequencing may present as episodic impairment of deglutition initiation, termed “freezing of swallowing” (Labeit et al., 2020a; Maetzler et al., 2016).

Beyond the basal ganglia, Lewy body pathology has also been identified in non-dopaminergic subcortical and cortical regions involved in the central control of swallowing (Warnecke et al., 2022). Dual-task studies show that swallowing performance deteriorates in participants with PD when performed concurrently with cognitively or motor demanding tasks (Labeit et al., 2021). This suggests that cognitive cortical reserve may compensate for OD but becomes compromised under increased cognitive load (Labeit et al., 2021) and may be further constrained by PD-associated neurodegeneration.

Beyond central involvement, peripheral mechanisms influence OD manifestations. Phosphorylated *α*-synuclein accumulates in both central nervous system and peripheral nerves, as seen in olfactory impairment, a common preclinical symptom of PD (Abraham et al., 2025). In oropharyngeal dysfunction, *α*-synuclein aggregates have been identified in pharyngeal motor and sensory nerves, mucosa, muscles, and salivary glands (Mu et al., 2024). This pathology may contribute to pharyngeal muscle atrophy and enlarged pharyngeal areas in patients with PD, which are associated with impaired swallowing functions (Curtis et al., 2020). Curtis et al. (2020) measured normalized pharyngeal area from VFSS in people with PD and healthy older adults, using two-dimensional fluoroscopic tracing techniques and examined relationships between pharyngeal area and swallowing kinematics, efficiency (bolus clearance ratio), and safety (Penetration-Aspiration Scale) in PD. They found that larger pharyngeal areas in PD were associated with reduced constriction, shorter airway closure, and decreased swallowing safety.

Moreover, reduced salivary substance P levels in patients with PD may underlie sensory deficits and impaired airway protection, as this neuropeptide is involved in regulation of the cough reflex (Schröder et al., 2019).

These pathophysiological mechanisms commonly culminate in pharyngeal bolus retention after swallowing, particularly in the valleculae, the most common OD phenotype in PD (Warnecke et al., 2021). Residue may spill over into the laryngeal vestibule, causing penetration or aspiration. Due to sensory impairments, patients often remain unaware of residue, preventing compensatory swallows. Other OD characteristics include delayed pharyngeal swallow initiation or premature spillage of the bolus into the pharynx resulting from impaired oral bolus containment (Warnecke et al., 2021). Impaired respiratory–swallow coordination may further increase airway invasion risks (Warnecke et al., 2023; Rangwala et al., 2023).

Oropharyngeal symptoms in PD vary across clinical motor phenotypes, suggesting benefits from phenotype-specific DBS adjustments, as with motor symptom control. A systematic review (Thijs and Dumican, 2023) reported that participants with tremor-dominant PD tend to exhibit less severe laryngeal symptoms, including dysphagia, than those with non-tremor dominant phenotypes, like postural instability and gait difficulty. However, it remains unclear whether the motor deficits affecting oropharyngeal function mirror those observed in the limbs. Dumican et al. (2024) found that oropharyngeal resting tremor significantly altered the timing of swallowing events, particularly shortening laryngeal vestibule closure duration and earlier airway reopening. These individuals demonstrated greater pharyngeal residue and more frequent aspiration/penetration, indicating that PD-related OD may involve distinct mechanisms in this subgroup requiring tremor-specific DBS programming.

Rigidity, another cardinal PD motor symptom, may also contribute to OD. A systematic review (Thijs and Dumican, 2023) showed that individuals with an akinetic-rigid phenotype had higher penetration/aspiration rates and more delayed pharyngeal swallow initiations than those with a tremor-dominant phenotype. Axial postural tone assessment showed that people with PD exhibit the most pronounced muscle tone increases in the cervical region (Franzén et al., 2009), with neck muscle tone 75% higher than healthy controls, exceeding trunk (22%) and hip (32%) increases. Given the close anatomical and functional relationship between cervical muscle tone and oropharyngeal structures, elevated neck rigidity may contribute to OD.

While rigidity may affect the structural aspects of swallowing, bradykinesia impacts its temporal dynamics. Pharyngeal bradykinesia is associated with vallecular residue, a primary characteristic of OD in PD (Warnecke et al., 2021) and was described to improve with levodopa-carbidopa intestinal gel (Labeit et al., 2020b). Bradykinesia of the tongue and mandible prolongs oropharyngeal transit time (Umemoto et al., 2011).

From a pathophysiological perspective, only certain components of OD, particularly those related to basal ganglia dysfunction such as tremorous, bradykinetic, rigid or freezing oropharyngeal motor patterns may be responsive to DBS. Other mechanisms, especially those involving non-dopaminergic or peripheral pathways, are less likely to be affected by DBS.

## 2 Current evidence on the effect of DBS on swallowing function

Studies were identified through database searches of MEDLINE, Embase and the Cochrane Library conducted in May 2025, using the search strategy: (‘parkinson disease’/exp. OR ‘parkinson*’) AND (‘deep brain stimulation’/exp. OR ‘deep brain stimulation’ OR ‘DBS’) AND (‘deglutition’/exp. OR ‘swallowing’/exp. OR ‘dysphagia’/exp. OR deglutition OR swallowing OR dysphagia). Inclusion criteria encompassed clinical studies including prospective and retrospective observational studies and case reports, investigating effects of DBS on swallowing function in people with PD. No language restrictions were applied; all identified papers were published in English. Duplicates were removed using database tools followed by manual checking. As this is a narrative review, screening was conducted by one reviewer (JH). In total, 24 studies were included in this review, encompassing 535 participants with PD. A systematic review methodology including risk of bias assessment or reviewer reliability procedures was not followed, which aligns with the intended scope of a narrative synthesis. Key aspects of included studies are summarized in Table 1.

**Table 1:** Characteristics and main findings of included studies examining deep brain stimulation effects on swallowing function in people with Parkinson’s disease.

Study	Study design	Target(s)	*N*	Objective swallowing outcome (outcome measure)	Subjective swallowing outcome (outcome measure)	DBS setting	Testing conditions	Key findings
Cebi et al. (2024)	RCT	Combined STN + SNr	20	FEES: Penetration/Aspiration (PAS) Swallow reflex (SOS) Pharyngeal residue (VAS) Oral preparation & transport (TOMASS) Functional level of oral intake of food & liquid (FOIS) Adverse events, e.g., coughing, asphyxia, bronchitis, aspiration pneumonia (patient diary)	Dysphagia associated quality of life (SWAL-QoL)	125 Hz 60 μs 0.5–2.0 mA All participants received concurrent swallowing therapy (3x1h group sessions per week for 8 weeks; strength-based exercises: Shaker-exercise, chin tuck- and tongue strengthening against resistance)	V0: MED-off & DBS-off/STN-on/SNr-on V1 (1–8 days after V0): MED-on & DBS-on V2 (8 weeks after V1): MED-on & DBS-on	No change in PAS score after intervention (STN vs. STN + SNr) (V1 vs. V2) Sig. Improvement in PAS scores only when groups are pooled (V1 vs. V2) Improvement in TOMASS* and pharyngeal residue* in STN group (V2) SOS score: delayed initiation of pharyngeal reflex* in STN group compared to STN-off (V0) SWAL-QOL: no change (STN vs. STN + SNr) Adverse events (2 mild, 1 severe)
Ciucci et al. (2008)	Prospective pre-post design	STN	14	VFSS: Pharyngeal transit time Maximal hyoid bone excursion Severity of impairment (Oral total composite score, Pharyngeal total composite score)	Not reported	not specified	V1: MED-off & DBS-on/DBS-off	Improvement of pharyngeal composite score* and pharyngeal transit time* in DBS-ON compared to DBS-OFF No change in oral parameters
Fagbami and Donato (2011)	Case report	STN	1	VFSS: Aspiration (not further defined) Pulmonary restriction (pulmonary function testing) Stridor Throat spasms, paroxysmal, non-productive cough	Patient report	Original settings (monopolar): Left: 130 Hz, 90 μs, and 1.6 V Right: 130 Hz, 60 μs, and 1.6 V Optimized settings (bipolar): Left: 160 Hz, 60 μs, and 1.5 V Right: 160 Hz, 60 μs, and 2.1 V	N/A	Stimulation-induced dysphagia and stridor Immediate resolvement of cough and dysphagia symptoms when DBS was turned off Subjective improvement by 80% in cough and swallowing
Henry et al. (2022)	Retrospective analysis	STN or GPi	54	VFSS: Penetration/aspiration (verbal description converted into PAS-score)	MDS-UPDRS	Not reported	V1 (pre-surgery): MED-on V2 (6 months post-surgery): MED-on & DBS-on	No sig. changes in converted PAS-scores after intervention, neither between groups (GPi vs. STN)
Kitashima et al. (2013)	Prospective pre-post design	STN	18	VFSS: Swallowing function (VF dysphagia scale) Oropharyngeal transit time, tongue movement, laryngeal elevation delay time	UPDRS	Not reported	V1 (pre-surgery): MED-on V2 (6 months post-surgery): MED-on & DBS-on/DBS-off	No change in VF dysphagia score (V1 vs. V2; V2), but changes in tongue movement* and laryngeal elevation delay time* (DBS on vs. off)
Krause et al. (2004)	Prospective pre-post design	STN	27	Not assessed	UPDRS	⌀ 148 Hz ⌀ 2.9 V ⌀ 73 μs	Testing conditions not explicitly stated V1 (pre-surgery) V2 (12 months post-surgery) Additional annual follow-ups, last assessment ⌀ 30 months post-surgery	Reported dysphagia as an adverse event in 3 participants
Krygowska-Wajs et al. (2016)	Prospective pre-post design	STN	20	Not assessed	UPDRS study-spefic questionnaire on gastro-intestinal dysfunction incl. dysphagia	130 Hz 1–2 V 60 μs	V1 (pre-surgery): MED-on V2 (3 months post-surgery): MED-on & DBS-on	Improvement in swallowing-related item of questionnaire* (V1 vs. V2)
Kulneff et al. (2013)	Prospective pre-post design	STN	11	FEES: Penetration/aspiration (PAS) Secretion status (secretion severity scale) Residues (scale for pharyngeal residue and clearance) Pre-swallow spillage (scale)	VAS for self-perception of swallowing	Not reported	V1 (pre-surgery): MED-off/MED-on V2 (6 months post-surgery): MED-on & DBS-on/DBS-off V3 (12 months post-surgery): MED-on & DBS-on/DBS-off	No changes in objective outcomes during FEES (any condition in V1 vs. V2) Improvement in subjective perception* (V1 MED-off vs. V2 MED-on & DBS-on; both in V2 & V3 in DBS-on vs. DBS-off)
Lengerer et al. (2012)	Retrospective analysis	STN	18	VFSS: Swallowing function (NZIMES Subscale One, Logemann-MBS-Parameters) Maximal hyoid bone excursion	Not reported	65–180 Hz 0.5–6.0 V 60–120 μs	V1 (pre-surgery): MED-on V2 (⌀ 20.3 months post-surgery): MED-on & DBS-on/DBS-off	Changes in: Reduced pharyngeal transit time* (V1 vs. V2) Reduced pharyngeal reaction time* (V1 vs. V2 DBS-on) Increased duration of cricopharyngeal opening* (V2, DBS-on vs. DBS-off) Reduced duration of cricopharyngeal opening* (V1 vs. V2 DBS-on & DBS-off) Increased time between cricopharyngeal opening and laryngeal vestibule closure* (V2, DBS-on vs. DBS-off)
Olchik et al. (2018)	Prospective pre-post design	STN	10	Non-instrumental assessment: Swallowing function (clinical evaluation, water swallow test, cervical auscultation, oximeter) Oral food intake (FOIS)	Not reported	Not reported	V1 (pre-surgery): MED-on V2 (6–12 months post-surgery): MED-on & DBS-on	No sig. changes in swallowing function
Pflug et al. (2020)	RCT	STN or combined STN + SNr	15	FEES: Pharyngeal residues (solids) (6-point scale) Penetration/aspiration (PAS) Leakage (scale for bolus location) Secretion (Murray score) Capability to swallow pills (4-point scale) Build-up phenomenon Swallowing duration (mixed consistencies)	Self-perception of swallowing (VAS)	STN: 125–130 Hz 1.5 V – 4.5 mA 60 μs STN + SNr: 125–130 Hz max. 5.2 mA 60 μs	V1: MED-on & DBS-off V2 (3 weeks after V1): MED-on & STN-on/STN + SNr-on V3 (3 weeks after V2): MED-on & STN-on/STN + SNr-on	No sig. changes (V1 vs. any DBS-on in V2 and V3) Simultaneous DBS-STN + SNr showed no added benefit compared to DBS-STN No sig. influence of DBS mode on subjective perception
Robertson et al. (2011)	RCT	STN or GPi	27	Mandibular movement (kinesiograph): Jaw velocity Voluntary jaw movement Automatic jaw movement	UPDRS	130–185 Hz ⌀3.28 V 60–150 μs	V1 (pre-surgery): MED-on/MED-off V2 (6 months post-surgery): MED-off & DBS-off/DBS-on; MED-on & DBS-off/DBS-on	Reduced velocity* in STN group compared to baseline (all conditions in V1 vs. V2) Increased velocity* in GPi group compared to baseline (V1 MED-off vs. V2 MED-off & DBS-off) No sig. change in velocity in GPi group in comparison V1 MED-on vs. V2 MED-on & DBS-on
Silbergleit et al. (2012a)	Prospective pre-post design	STN	14	VFSS: Penetration/aspiration (4-point scale) Oral phase characteristics, pharyngeal phase characteristics (4-point scale)	Self-perception of swallowing (DHI)	Not reported	V1 (pre-surgery): MED-on/MED-off V2 (3 months post-surgery): MED-on & DBS-on/DBS-off; MED-off & DBS-on/DBS-off V3 (12 months post-surgery): MED-on & DBS-on/DBS-off; MED-off & DBS-on/DBS-off	Improvements* in subjective evaluation (DHI) (V1 vs. V2; V1 vs. V3) Improved oral preparation of thin liquids* (V3: MED-off & DBS-on vs. MED-off & DBS-off) Improved swallowing response for solids* (V3: MED-off & DBS-on vs. MED-off & DBS-off)
Smith-Hublou et al. (2024)	Retrospective cross-sectional observational study	GPi	36	VFSS: Swallowing safety (PAS, DIGEST) Swallowing timing measures Relationships between swallowing safety and DBS settings	Not reported	Unilateral GPi-DBS (⌀) Left: 155.83 Hz 2.76 V 88.33 μs Right: 168.75 Hz 2.64 V 86.25 μs Bilateral GPi-DBS (⌀) Left: 151.56 Hz 2.76 V 91.88 μs Right: 152.81 Hz 2.68 V 86.25 μs	Pre-surgical evaluation: MED-on Post-surgical evaluation: (⌀ 6.7–7.5 months post-surgery): unilateral-DBS-on & MED-on; bilateral-DBS-on & MED-on	No changes in pre- vs. post-surgical evaluation No changes between DBS modalities (unilateral vs. bilateral GPi-DBS) No associations between swallowing safety and stimulation frequency or pulse width
Sundstedt et al. (2017a)	Prospective pre-post design	cZi	14	FEES: Penetration/aspiration (PAS) Secretion status (Secretion severity scale) Pre-swallow spillage, pharyngeal residue, pharyngeal clearance (rated as present/absent)	UPDRS	125–160 Hz	V1 (pre-surgery): MED-on V2 (6 months post-surgery): MED-on & DBS-on/DBS-off V3 (12 months post-surgery): MED-on & DBS-on/DBS-off	No sig. changes in any of the investigated outcomes pre- to post-surgery
Sundstedt et al. (2017b)	Prospective pre-post design	cZi	9	Not assessed	Swallow-related Quality of Life (SWAL-QoL, VAS)	125–160 Hz	V1 (pre-surgery): MED-on V2 (12 months post-surgery): MED-on & DBS-on	No sig. changes in swallow-related quality of life (V1 vs. V2)
Sundstedt et al. (2012)	Prospective pre-post design	cZi	8	FEES: Penetration/aspiration (PAS) Secretion status (Secretion severity scale) Preswallow spillage, pharyngeal residue, pharyngeal clearance (rated as present/absent)	Swallow-related quality of life (non-validated questionnaire, VAS)	Not reported	V1 (pre-surgery): MED-on/MED-off V2 (6 months post-surgery): MED-on & DBS-on/DBS-off V3 (12 months post-surgery): MED-on & DBS-on/DBS-off	Reduced pre-swallow spillage* (V1 MED-on vs. V2 both in DBS-off and DBS-on) Increased pre-swallow spillage* (V3 at DBS-on vs. DBS-off) (no sig. Difference to V1 MED-on state) No sig. changes in subjective outcomes
Troche et al. (2014)	Retrospective analysis	STN or GPi	33	VFSS: Penetration/aspiration (PAS)	Swallow-related quality of life (SWAL-QOL) UPDRS	Not reported	V1 (pre-surgery): MED-on V2 (6 months post-surgery): MED-on & DBS-on	No changes in PAS-scores in GPi-DBS group (V1 vs. V2) Increase in PAS-score* after STN-DBS (V1 vs. V2) No change in swallowing-related quality of life pre- to post-surgery in neither group (V1 vs. V2)
Troche et al. (2016)	Case report	Combined right STN + left GPi	1	VFSS Swallowing function Laryngoscopy: Airway protective outcomes	Not reported	Optimized settings: 135 Hz 3.8 V 90 μs	N/A	Immediate improvements in swallowing safety and airway protective outcomes with optimized DBS settings
Wolz et al. (2012)	Prospective pre-post study	STN	34	Not reported	Water swallow test (200 mL) if swallowing difficulties were observed, severity was rated by researcher on a VAS	Not reported	V1 (at least 9 months post-surgery): MED-off & DBS-on/DBS-off	Subjective improvement* in DBS-on vs. DBS-off state
Xie et al. (2015)	Prospective pre-post design	STN	7	VFSS: Frequency of aspiration (PAS)	Perceived swallowing difficulty (SDQ)	60 Hz/130 Hz/ Off Left: 60 Hz ⌀3.2 ± 0.4 V ⌀90.0 ± 24.5 μs Right: 60 Hz ⌀3.1 ± 0.4 V ⌀81.4 ± 14.6 μs	V1: MED-on & DBS-60-Hz/130 Hz/off V2 (⌀ 6 weeks after V1): MED-on & DBS-60 Hz	Reduced aspiration frequency* with 60 Hz vs. 130 Hz (within V1) Reduced perceived swallowing difficulty* with 60 Hz vs. 130 Hz (within V1)
Xie et al. (2018)	Prospective pre-post design	STN	11	VFSS: Frequency of aspiration (PAS)	Perceived swallowing difficulty (SDQ)	60 Hz/130 Hz/ Off Left: 60 Hz ⌀3.0 V ⌀76.0 μs Right: 60 Hz ⌀2.9 V ⌀68.0 μs	V1: MED-on & DBS-60-Hz/130 Hz/off V2 (⌀14,5 months after V1): MED-on & DBS-60 Hz/130 Hz/off	Short-term: Reduced frequency of aspiration* with 60 Hz vs. 130 Hz (no change in 60 Hz vs. DBS-off) (within V1) Reduced perceived swallowing difficulty* with 60 Hz vs. DBS-off (within V1) Long-term: No benefit of 60 Hz in frequency of aspiration or perceived swallowing difficulty (V1 vs. V2)
Xu et al. (2018)	Retrospective analysis	STN	80	Not reported	UPDRS	Tremor-dominant type: 163.0 ± 20.9 Hz 2.9 ± 0.5 V 86.0 ± 10.6 μs Akinetic-rigid type: 174.2 ± 9.4 Hz 2.9 ± 0.4 V 87.9 ± 12.1 μs Mixed type: 168.5 ± 13.4 Hz 3.1 ± 0.4 V 87.2 ± 12.0 μs	V1 (pre-surgery): MED-on/MED-off V2 (⌀ 4.9 years post-surgery): MED-on/MED-off & DBS-on	No long-term benefit of STN-DBS Swallowing* deteriorated (V1 vs. V2)
Zibetti et al. (2007)	Prospective pre-post design	STN	36	Not reported	UPDRS	At 12 months: 136 ± 14.8 Hz 3.2 ± 0.4 V 63.3 ± 9.5 μs At 24 months: 136.1 ± 12.5 Hz 3.3 ± 0.3 V 65.0 ± 11.3 μs	V1 (pre-surgery): MED-on/MED-off V2 (12 months post-surgery) MED-on/MED-off & DBS-on V3 (24 months post-surgery) MED-on/MED-off & DBS-on	Improved swallowing* (V1 MED-off vs. V2 & V3 MED-on & DBS-on)

This table presents study characteristics including anatomical target, sample sizes, and swallowing outcomes (instrumental and subjective). DBS parameters and key findings are summarized for each study. Asterisks (*) indicate statistically significant results, with *p* < 0.05. Study visits are coded as: V0 (screening), V1 (baseline), V2 + (follow-up measurements). DBS-on, active stimulation; DBS-off, inactive stimulation; DHI, dysphagia handicap index; FEES, flexible endoscopic evaluation of swallowing; FOIS, Functional oral intake scale; GPi, globus pallidus internus; MED-on, medication-on state; MED-off, medication-off state; NZIMES, New Zealand Index for Multidisciplinary Evaluation of Swallowing; PAS, penetration aspiration scale; SDQ, Swallowing Disturbance Questionnaire; SNr, substantia nigra; SOS, site of swallow reflex initiation; STN, subthalamic nuleus; SWAL-QoL, Swallowing Quality of Life Questionnaire; TOMASS, Test of Mastication and Swallowing Solids; UPDRS, Unified Parkinson’s Disease Rating Scale; VAS, visual analog scale; VF dysphagia scale, Videofluoroscopic dysphagia scale; VFSS, videofluoroscopic swallow study.

The included studies employed diverse methodological approaches to evaluate swallowing function. Instrumental assessment was performed in *n* = 16 studies, with VFSS used in *n* = 11 studies and FEES in *n* = 5 studies. The most commonly reported outcome measure used for instrumental assessments was the Penetration-Aspiration Scale (PAS; *n* = 10), a validated 8-point ordinal scale that rates the depth of airway invasion and the patient’s response to material entering the airway (Rosenbek et al., 1996) (see Table 2). Other scales applied include the Dynamic Imaging Grade of Swallowing Toxicity [DIGEST (Hutcheson et al., 2017); *n* = 1] and the Secretion-Severity Scale (Murray et al., 1996); (*n* = 3). Patient-reported outcome measures included quality-of-life questionnaires (Swallowing Quality of Life Questionnaire [SWAL-QoL (McHorney et al., 2002); *n* = 3]) and Dysphagia Handicap Index [DHI (Silbergleit et al., 2012b); *n* = 1]). Separately, *n* = 3 studies reported the dysphagia-related questions of the Unified Parkinson’s Disease Rating Scale (UPDRS) (Goetz et al., 2008; Fahn et al., 1987), a general motor assessment tool while *n* = 2 studies employed the Swallowing Disturbance Questionnaire (SDQ) (Manor et al., 2007). A minority of studies used investigator-reported outcome measures including clinical bedside evaluation (*n* = 1), a water swallow test (*n* = 1), the Test of Mastication and Swallowing Solids [TOMASS (Huckabee et al., 2018); *n* = 1] and the Functional Oral Intake Scale [FOIS (Crary et al., 2005); *n* = 2]. Kinesiography and laryngoscopy were applied by one study, respectively.

**Table 2:** The Penetration-Aspiration Scale (Rosenbek et al., 1996).

PAS-score	Description
1	Material does not enter the airway.
2	Material enters the airway, remains above the vocal folds, and is ejected from the airway.
3	Material enters the airway, remains above the vocal folds, and is not ejected from the airway.
4	Material enters the airway, contacts the vocal folds, and is ejected from the airway.
5	Material enters the airway, contacts the vocal folds, and is not ejected from the airway.
6	Material enters the airway, passes below the vocal folds and is ejected into the larynx or out of the airway.
7	Material enters the airway, passes below the vocal folds, and is not ejected from the trachea despite effort.
8	Material enters the airway, passes below the vocal folds, and no effort is made to eject.

The following section provides a structured overview of included studies, organized by DBS targets, patient-reported outcome measures and stimulation settings.

### 2.1 DBS targets and mechanisms on swallowing

For PD motor symptom management, the STN and GPi represent common DBS targets. European treatment guidelines (Deuschl et al., 2022) indicate no evidence-based preference for either target. Among included papers, 17 investigated DBS in STN (Ciucci et al., 2008; Troche et al., 2014; Henry et al., 2022; Xie et al., 2018; Xie et al., 2015; Krause et al., 2004; Fagbami and Donato, 2011; Kitashima et al., 2013; Krygowska-Wajs et al., 2016; Kulneff et al., 2013; Lengerer et al., 2012; Olchik et al., 2018; Robertson et al., 2011; Silbergleit et al., 2012a; Wolz et al., 2012; Xu et al., 2018; Zibetti et al., 2007), four in GPi (Smith-Hublou et al., 2024; Troche et al., 2014; Henry et al., 2022; Robertson et al., 2011) and three in caudal zona incerta (cZI) (Sundstedt et al., 2017a; Sundstedt et al., 2017b; Sundstedt et al., 2012). Two studies employed combined STN and substantia nigra (SNr) DBS (Cebi et al., 2024; Pflug et al., 2020), while one utilized combined STN + GPi-DBS (Troche et al., 2016).

#### 2.1.1 STN-DBS

STN-DBS is known to modulate both direct and indirect motor pathways (Cavallo et al., 2025), including excitatory glutamatergic projections to the substantia nigra pars compacta (which influences dopaminergic output) and loops involving the brainstem (Freestone et al., 2015).

Of 24 identified studies, eight clinical studies (Henry et al., 2022; Xie et al., 2018; Kitashima et al., 2013; Kulneff et al., 2013; Lengerer et al., 2012; Olchik et al., 2018; Silbergleit et al., 2012a; Zibetti et al., 2007) reported no significant changes in swallowing function after STN-DBS. Two studies reported positive effects of STN-DBS (Ciucci et al., 2008; Xie et al., 2015), while four studies (Troche et al., 2014; Krause et al., 2004; Robertson et al., 2011; Xu et al., 2018) described detrimental effects on swallowing.

##### 2.1.1.1 Studies reporting positive or neutral STN-DBS outcomes

Using VFSS in 14 participants, Ciucci et al. (2008) observed significant improvements in pharyngeal transit time and pharyngeal composite scores (encompassing swallowing safety, efficiency, and structural movement parameters) when comparing DBS-on with DBS-off in the medication-off state. Hyoid bone excursion and oral stage parameters remained unchanged. Xie et al. (2015) investigated the impact of stimulation frequency on swallowing function in bilateral STN-DBS by comparing DBS-off, 60 Hz, and 130 Hz conditions in the medication-on state (*n* = 7). They observed during VFSS that 60 Hz significantly reduced aspiration frequency and subjective swallowing difficulty compared to 130 Hz. However, no statistically significant difference was found when comparing 60 Hz stimulation with DBS-off, suggesting that DBS with 60 Hz does not inherently improve swallowing. Given the small sample size, this study was likely underpowered, impacting statistical analyses. Silbergleit et al. (2012a) evaluated swallowing during VFSS across multiple medication and stimulation states (MED-on/off, DBS-on/off) at both 3 and 12 months. Improvements in oral preparation of thin liquids and pharyngeal response for solids were observed at 12 months post-surgery in MED-off/DBS-on conditions compared to MED-off/DBS-off, while global penetration/aspiration-based safety outcomes remained unchanged. Lengerer et al. (2012) conducted VFSS with detailed timing measures (pharyngeal transit, reaction time, cricopharyngeal opening). Several timing parameters improved post-surgery in DBS-on vs. DBS-off conditions, although the retrospective design, heterogeneous DBS settings, and variable follow-up duration limit causal inferences. Moreover, Kitashima et al. (2013) investigated swallowing function in DBS-on vs. DBS-off conditions and found no changes in videofluoroscopic dysphagia score between visits. However, significant changes in tongue movement and laryngeal elevation delay time were observed when comparing DBS-on vs. DBS-off states. Kulneff et al. (2013) applied a comprehensive FEES protocol including PAS, secretion status, residue scoring and pre-swallow spillage after STN-DBS. For statistical analysis, all scales were transformed so the lowest score was 0 and the mean of each scale was derived. Although no changes were detected during FEES post-surgery, patients consistently reported improved subjective swallowing.

##### 2.1.1.2 Studies reporting negative STN-DBS outcomes

Other studies reported negative effects of STN-DBS on swallowing. Troche et al. (2014) retrospectively analyzed the effects of unilateral STN-DBS on swallowing safety in 14 individuals with PD, comparing PAS scores before and 6 months after surgery. The baseline assessment was conducted in the medication-on state, while the post-surgical evaluation was performed in medication-on and DBS-on state. The study found a significant increase in PAS scores postoperatively, indicating a decline in swallowing safety. Beyond pharyngeal swallowing function, one study examined the impact of STN-DBS on jaw movements. Robertson et al.’s (2011) research team conducted a prospective intervention study to evaluate the effects of DBS target site on bradykinetic jaw movements in participants with STN-DBS (*n* = 14). They assessed mandibular movement through kinesiographic measurements and oromotor tasks. A chin-mounted magnet tracked by eyeglass sensors provided 3D mandibular data. Baseline assessments prior to surgery included both medication-off and -on states. The post-surgery testing included four different conditions: (1) medication-off and DBS-off; (2) medication-on and DBS-off (3) medication-off and DBS-on; (4) both medication-on and DBS-on. The authors reported a significant reduction in jaw velocity in the STN-DBS group in all conditions compared to medication-off in the baseline assessment. However, swallowing efficiency or safety were not assessed directly.

Krause et al. (2004) reported OD as an adverse event in 3 out of 27 participants in a study investigating the long-term effects of bilateral STN-DBS on overall motor symptoms in PD. However, neither a definition of OD nor criteria for adverse event classification were specified, limiting interpretation of these findings.

##### 2.1.1.3 STN-DBS mechanisms

**Figure 1:**
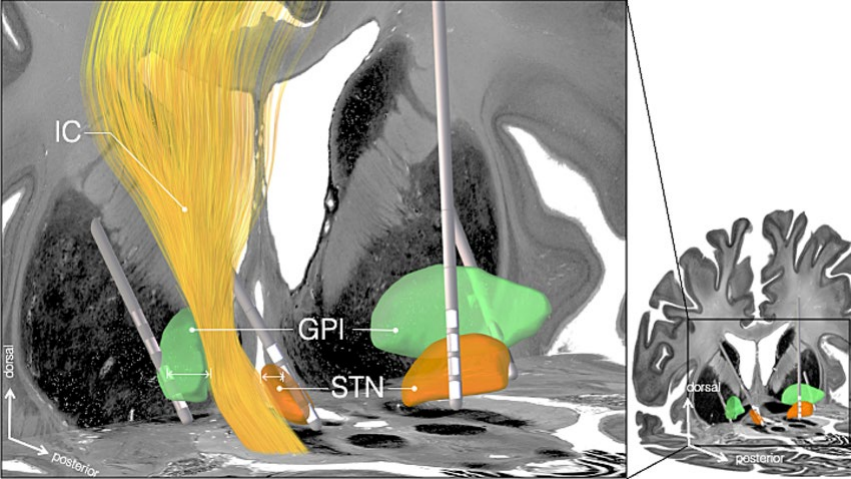
Deep brain stimulation electrode placement in the basal ganglia showing subthalamic nucleus and globus pallidus internus targets in relation to the internal capsule. Anatomical structures are depicted based on the BigBrain atlas (Amunts et al., 2013). DBS leads show typical positioning for therapeutic stimulation.

While STN-DBS mechanisms often produce motor improvement, the STN lies adjacent to the internal capsule, especially with its lateral aspect (see Figure 1). If stimulation spreads toward the capsule with its corticobulbar fibers, which descend through the genu of the internal capsule and control facial, lingual, and pharyngeal muscles, this can result in capsular side effects such as dysarthria (Jergas et al., 2025), facial pulling (Mahlknecht et al., 2017), or potentially OD. This phenomenon may explain why some patients show negative effects on swallowing, especially in the medication-on state when STN-DBS and pharmacological pathways interact.

#### 2.1.2 GPi-DBS

As the principal output nucleus of the basal ganglia, the GPi sends GABAergic inhibitory projections to the thalamus and brainstem, both key regions involved in motor control, including bulbar function. In PD, overactivity in the GPi is considered to contribute to disrupted motor signaling (Dostrovsky et al., 2001). DBS targeting the GPi may reduce this pathological overactivity, thereby improving the transmission of motor signals to brainstem centers such as the swallowing central pattern generator (CPG), potentially modulating swallowing function (Au et al., 2021). The swallowing CPG, a neural network located in the brainstem, coordinates the sequential motor patterns required for swallowing and receives modulatory input from higher motor centers including basal ganglia pathways (Wei et al., 2024). None of the four included studies investigating the impact of GPi on swallowing function found significant changes pre- to post-surgery. One of these studies was a prospective clinical trial (Robertson et al., 2011), while three studies were retrospective chart reviews (Smith-Hublou et al., 2024; Troche et al., 2014; Henry et al., 2022). The prospective trial by Robertson et al. (2011) found that compared with baseline medication-off conditions, the GPi group (*n* = 13) showed significant postoperative improvements in the DBS- and medication-off condition, with increased jaw opening and closing velocity (both *p* < 0.05), and faster thin-elicited biting velocity (defined as the final, consistent peak velocity of the jaw, measured when the participant bites through a thin piece of raw carrot in a single, ballistic motion; *p* < 0.01), although their performance during combined DBS- and medication-on was not different from medication-on pre-surgery. These findings indicate that GPi-DBS may have produced persistent improvements in jaw function which could reflect several mechanisms including microlesion effect, as commonly observed in DBS. The absence of additional benefit when stimulation is active may reflect ceiling effects with medication. Smith-Hublou et al. (2024) retrospectively assessed swallowing in 36 participants in a cross-sectional observational study with some overlapping participants. The team used PAS for swallowing safety and DIGEST (Hutcheson et al., 2017) for both safety and efficiency, and timing parameters including pharyngeal transit time and laryngeal approximation reaction time. The primary outcome was the percentage of thin-liquid bolus trials rated “unsafe,” defined as PAS scores of ≥3. No effect of GPi-DBS was found on any outcome. Similar findings were reported by Henry et al. (2022), who investigated 25 participants with bilateral GPi-DBS pre- and 6-months post-surgery and retrospectively reported their results. In this retrospective study, the researchers converted the verbal description from the report into a PAS score, a post-hoc scoring approach that may have introduced validity concerns. No significant difference in PAS score was detected when compared to baseline or to the other study group receiving STN-DBS (*n* = 29). In contrast to the worsened PAS scores observed after STN-DBS, participants receiving GPi-DBS (*n* = 19) in the study by Troche et al. (2014) showed no significant change in PAS scores at 6 months post-surgery.

##### 2.1.2.1 GPi-DBS mechanisms

The findings described above align with the anatomical considerations for GPi targeting, as GPi-DBS is generally located at a safer distance from the internal capsule (see Figure 1), reducing the likelihood of corticobulbar side effects from stimulating the capsule that could impair swallowing. However, the larger anatomical size of the GPi may cause greater variability in electrode placement than the more compact STN. With only one study reporting precise GPi coordinates (Robertson et al., 2011), direct comparisons and identification of an optimal stimulation site to target OD were not possible (see Supplementary Table 1 for reported coordinate data). Furthermore, the larger dimensions of the GPi also require higher charge densities (Au et al., 2021), so electrodes near the internal capsule may induce corticobulbar side effects at lower thresholds due to increased pulse rates and amplitudes.

#### 2.1.3 cZI-DBS

The cZI has widespread connections to cortical, thalamic, and brainstem regions, including those involved in autonomic and bulbar functions (Lau et al., 2020). Although not as well studied, stimulation in this region may modulate both dopaminergic and brainstem networks, potentially providing symptom relief with fewer capsular side effects due to its relative distance from the internal capsule (Mai and Paxinos, 2012).

One research group investigated bilateral cZI-DBS in a prospective pre-post stimulation design and published findings on swallowing function and swallowing related quality of life across three different studies (Sundstedt et al., 2017a; Sundstedt et al., 2017b; Sundstedt et al., 2012), with participant overlap between studies. The 2017 swallowing safety study (Sundstedt et al., 2017a) (*n* = 14) constituted an extended analysis of the 2012 study (Sundstedt et al., 2012) (*n* = 8), examining the same subset. Overall, the study team found no changes in swallowing related quality of life, swallowing safety measured during FEES by PAS and the Secretion Severity Scale (Murray et al., 1996) nor in pre-swallow spillage, pharyngeal residue and pharyngeal clearance which were rated dichotomously (present/absent) (Sundstedt et al., 2017a; Sundstedt et al., 2012).

#### 2.1.4 Simultaneous DBS in different targets

Three studies reported on simultaneous DBS of different anatomical targets. Two of these were randomized double-blind clinical trials investigating combined DBS of STN and SNr.

The SNr connects to the pedunculopontine nucleus, superior colliculus, and the swallowing CPG, making it an intriguing target for bulbar symptom modulation (Wichmann and Delong, 2011). Stimulation here may inhibit excessive neural firing and restore normal function in downstream brainstem networks. Importantly, the SNr is located further from the internal capsule, reducing the risk of corticospinal or corticobulbar activation and associated swallowing side effects (Mai and Paxinos, 2012).

Cebi et al. (2024) compared the effects of STN-DBS versus combined STN + SNr-DBS in 20 participants with PD who had previously received STN-DBS for at least 6 months prior to study enrollment in a double-blind randomized controlled trial. Following baseline assessment under existing STN-DBS settings, participants were randomized to receive either continued STN-DBS or combined STN + SNr-DBS for 8 weeks before post-intervention evaluation. All participants received concurrent swallowing therapy consisting of group sessions (three times per week for 8 weeks) with strength-based exercises (Shaker-exercise, chin tuck against resistance and tongue strengthening against resistance), precluding attribution of effects to DBS alone. The researchers conducted a power analysis determining that 10 participants per group would provide 81% power to detect significant PAS score changes and performed an intention-to-treat analysis on all 20 enrolled participants despite six drop-outs. The research group found no significant difference in PAS scores obtained during FEES when comparing STN-DBS with combined STN + SNr-DBS. A significant effect was only detected after pooling the data and comparing baseline with follow-up measurements. The study group used the median and interquartile ranges for statistical analysis of the PAS. Similarly, Pflug et al. (2020) compared STN + SNr-DBS with conventional STN-DBS. Using a randomized, double-blind crossover study design, 15 participants with PD underwent baseline assessment followed by either STN-DBS or combined STN + SNr-DBS for 3 weeks, with assessments conducted after each treatment period. Likewise, they did not find any positive effects on pharyngeal residue and penetration−/aspiration events after simultaneous STN + SNr-DBS. Of the 15 included participants, four dropped out due to adverse events after starting combined STN + SNr-DBS. Although no significant group-level effects were observed, individual outcomes varied considerably compared to DBS-off: three participants showed worsening PAS scores for water under both stimulation modes, and two demonstrated improvements. In this study, PAS scores were grouped into three categories for analysis: “non-pathological” (PAS 1–2), “laryngeal penetration” (PAS 3–5), and “aspiration” (PAS 6–8). While Pflug et al. (2020) determined through power analysis that 11 participants provided sufficient statistical power for their cross-over design, the authors acknowledged that this small sample size could still limit detection of group-level differences in swallowing function. In a case report, Troche et al. (2016) described a patient with VFSS-confirmed OD and bilateral DBS (STNand GPi). Airway protection improved after reprogramming, with final settings of 135 Hz, 120 μs, 2.9 V (GPi) and 135 Hz, 90 μs, 3.8 V (STN). Interpretation remains limited since baseline parameters were not provided.

### 2.2 Patient-reported outcome measures

Several studies used patient-reported outcome measures to evaluate swallowing. Some studies assessed swallowing using the UPDRS. One retrospective study of 80 participants examined the long-term effects of bilateral STN-DBS across Parkinson’s disease motor subtypes and reported a decline in swallowing function after a mean follow-up of 4.9 years (Xu et al., 2018). In a study on long-term motor outcomes following bilateral STN-DBS, Krause et al. (2004) described a worsening in mean swallowing scores from baseline to one year post-DBS surgery (0.3 vs. 1.1, respectively), though statistical significance of this difference was not reported. Zibetti et al. (2007) observed significant improvements in swallowing at 12 and 24 months post-surgery in 36 participants in the MED-on state with STN-DBS compared with the pre-surgical MED-off state. However, no significant differences were found when comparing the pre-operative MED-on state to the post-surgical DBS-ON & MED-on states at 12 or 24 months. Therefore, the observed improvements cannot be solely attributed to DBS and may reflect medication effect. Overall, results derived from the UPDRS should be interpreted with caution, as the scale includes only a limited number of swallowing-related items and was not designed to provide a comprehensive or sensitive assessment of swallowing function or OD severity.

Krygowska-Wajs et al. (2016) used a study-specific gastrointestinal dysfunction questionnaire containing a single OD item rated on a 5-point severity scale. At 3 months post STN-DBS surgery, fewer participants reported OD, and mean severity scores decreased significantly. Xie et al. (2015) reported that 60-Hz stimulation reduced perceived swallowing difficulty by 80% compared to 130 Hz in their 2015 study, though their 2018 study (Xie et al., 2018) found this subjective benefit was not sustained at long-term follow-up at 14.5 months.

Visual analog scales were employed in three studies. Pflug et al. (2020) assessed self-perceived swallowing function and found no effect of stimulation target (STN vs. STN + SNr). Kulneff et al. (2013) evaluated participants’ perceived percentage deterioration in swallowing and reported significantly improved swallowing during STN-DBS-on compared with STN-DBS-off conditions, while no significant effect was detected on FEES assessment. Sundstedt et al. (2017b) found no clinically significant negative impact of cZI-DBS on participants’ self-perceived swallowing function.

Three studies used the SWAL-QOL to assess swallowing-related quality of life. Troche et al. (2014) reported no pre- to post-surgical differences in either GPi-DBS or STN-DBS groups, despite significantly worsened mean PAS-scores in the STN-DBS group. Similarly, Sundstedt et al. (2017b) found no change following cZI-DBS, and Cebi et al. (2024) observed no clinically relevant changes pre- to post-surgery or between STN-DBS and STN + SNr-DBS groups. In contrast, Silbergleit et al. (2012a) reported a significant improvement in DHI scores following STN-DBS.

In summary, patient-reported outcomes yielded inconsistent findings across studies. While validated, swallowing-specific quality-of-life measures generally indicated no deterioration following DBS, results derived from broader motor assessments were more variable.

### 2.3 DBS stimulation settings and mechanisms

The impact of DBS stimulation settings on swallowing in PD has been investigated only rarely, and stimulation parameters were often incompletely reported. None of the included studies systematically adjusted settings based on swallowing outcomes, except for two case reports (Fagbami and Donato, 2011; Troche et al., 2016). Overall, swallowing function was generally not considered in DBS programming.

#### 2.3.1 Stimulation parameters

DBS programming involves adjusting multiple stimulation parameters, including frequency (Hz), amplitude (V), and pulse width (μs), which can be individually optimized to maximize therapeutic benefit while minimizing adverse effects (Hartmann et al., 2019). Frequency settings have been investigated for their differential effects on motor symptom subtypes. Low-frequency stimulation (LFS, <100 Hz) may restore physiological gamma-oscillations, while high-frequency stimulation (HFS, >100 Hz, typically 130 Hz) suppresses pathological rhythms and is more effective for appendicular symptoms (Cavallo et al., 2025; Freestone et al., 2015). LFS may be more beneficial for axial symptoms such as gait (Jergas et al., 2025).

With respect to swallowing, Smith-Hublou et al. (2024) found no significant associations between swallowing safety (percentage of “unsafe thin-liquid swallows,” defined as PAS > 3) and stimulation parameters including frequency, pulse width, or voltage in their retrospective analysis of 36 participants with GPi-DBS. In contrast, Xie et al. (2015) observed reduced aspiration frequency and improved self-reported swallowing difficulty under low-frequency STN-DBS (60 Hz) compared to HFS (130 Hz). Aspiration frequency was calculated by summing the number of swallows with a PAS rating of ≥ 6. These effects were only seen in a direct comparison and after 6–8 weeks of LFS however were not sustained at 14.5 months (Xie et al., 2018). Both studies by Xie et al. conducted power analyses but were underpowered due to lower recruitment, potentially limiting the magnitude of detected effects. Notably, participants had medication-refractory freezing of gait that improved under LFS, suggesting that alleviation of oropharyngeal freezing may have contributed to swallowing benefits. Therefore, findings may not be generalizable to the broader PD population. Moreover, stimulation voltage was not adjusted when switching from HFS to LFS, reducing total electrical energy delivered (TEED) and complicating interpretation. TEED represents the cumulative energy transferred during stimulation and is a function of the stimulation frequency, pulse width, voltage and the impedance between electrode and surrounding brain tissue (Koss et al., 2005). TEED is proportional to the square of voltage. Therefore, maintaining constant voltage across different frequencies results in different total energy exposures, making it difficult to determine whether observed effects are due to frequency changes per se or to differences in TEED.

Given the limited body of literature specifically addressing swallowing outcomes, insights from related domains such as speech and voice may provide useful contextual information. Studies in these areas demonstrate highly variable effects, with high-frequency or high-amplitude stimulation often worsening speech and articulation, while lower frequencies or shorter pulse widths may offer some benefit (Skodda, 2012; Tabari et al., 2024); the anatomical target is also critical, as pallidal stimulation can improve voice quality but may worsen other speech features while other targets (STN, Thalamic, Zona Incerta) are more often linked to deterioration of vocal parameters (Baudouin et al., 2023).

#### 2.3.2 Lead configuration: monopolar vs. bipolar DBS

Lead configuration strongly influences DBS effects. Monopolar stimulation produces a broad electric field and increases the risk of unintended activation of corticobulbar fibers in the internal capsule, which are essential for speech and swallowing and may underlie stimulation-induced dysarthria in STN-DBS (Wei et al., 2024; Jergas et al., 2025; Mahlknecht et al., 2017; Liégeois et al., 2016). Bipolar stimulation generates a more confined electric field, thereby reducing current spread. Recruitment also depends on the alignment between the electric field and fiber orientation. With cylindrical contacts, monopolar or simple bipolar configurations tend to bias activation toward fibers parallel to the lead (Slopsema et al., 2018; Butenko et al., 2025), further increasing the risk of corticobulbar involvement. In contrast, multipolar or directional stimulation can bias recruitment toward therapeutic pathways such as the hyperdirect tract (Slopsema et al., 2018; Butenko et al., 2025), and anatomy-guided programming may thus help clinicians reduce bulbar side effects.

Eight studies reported stimulation configuration: some applied mixed settings within individuals (Xie et al., 2018; Xie et al., 2015; Xu et al., 2018; Zibetti et al., 2007), while others included monopolar or bipolar stimulation exclusively (Lengerer et al., 2012; Zibetti et al., 2007; Cebi et al., 2024). Fagbami and Donato (2011) provided a clear case example: a patient developed OD with aspiration, stridor, and respiratory restriction under monopolar STN-DBS (130 Hz, 90/60 μs, 1.6 V bilaterally). When DBS was suspended, symptoms improved and aspiration resolved; subsequent reprogramming to bipolar stimulation (160 Hz, 60 μs, 1.5–2.1 V) achieved sustained clinical improvement. The improvement may be attributed to reduced current spread to corticobulbar fibers.

#### 2.3.3 Stimulation directionality: omnidirectional vs. directional electrodes

Electrode design further affects DBS outcomes. In a homogeneous medium, traditional omnidirectional leads generate axially symmetric electric fields, which can spread into surrounding structures, whereas segmented leads allow directional current steering. Directional stimulation reduces the risk of activating corticospinal and corticobulbar tracts, which is especially relevant near the highly myelinated internal capsule, where current distribution can be altered (Ineichen et al., 2018). Steering current away from these tracts may reduce stimulation-induced side effects such as OD. Various commercial and non-commercial methods are available to predict which brain tissue will be affected by DBS stimulation and to estimate the volume of neural activation.

#### 2.3.4 Tailored optimization

In addition to frequency and configuration, individualized optimization of stimulation settings has been reported in case studies (Fagbami and Donato, 2011; Troche et al., 2016). Similar to the previously outlined case study by Fagbami and Donato (2011), Troche et al. (2016) described improved airway protection in a patient with bilateral DBS (STN and GPi) after tailored reprogramming, although specific stimulation parameter adjustments were not provided. Such cases illustrate the potential of individualized DBS programming to modulate swallowing outcomes, even though systematic protocols are currently lacking.

## 3 Discussion

In total, 24 studies were included: three randomized double-blind trials, 14 prospective pre–post studies, five retrospective analyses, and two case reports. Some compared anatomical targets, while others examined stimulation settings, most commonly assessing pre- versus post-surgery or DBS-on versus -off. Many studies were open-label and/or retrospective in design, with small sample sizes. Additional limitations included suboptimal outcome measures for swallowing, as well as incomplete reporting of swallowing protocols, stimulation parameters, and medication status, which further compromised comparability across studies.

A major limitation across studies is the consistently small sample size, with power analyses only reported in four (Xie et al., 2018; Xie et al., 2015; Cebi et al., 2024; Pflug et al., 2020) of the 24 studies. As a result, most investigations were likely underpowered to detect clinically meaningful effects. This issue is further compounded by the heterogeneity of DBS effects, which can vary substantially within individuals. Without adequate sample sizes or stratification by OD phenotype, such variability is difficult to capture at the group level, limiting the interpretability and generalizability of findings.

Notably, four studies published between 2020 and April 2025 included two double-blind randomized controlled trials and two retrospective studies. All four utilized instrumental OD assessments, representing methodological improvement over earlier studies. However, participant selection may have introduced bias, as most individuals had no or only minimal swallowing safety impairment at baseline, based on reported PAS scores. Consequently, potential for observing treatment-related improvement was limited, and generalizability to broader clinical populations, particularly patients with severe OD remains restricted. However, most of these studies also examined outcomes beyond swallowing safety, measuring timing parameters such as pharyngeal swallow phase (Smith-Hublou et al., 2024), mastication (Cebi et al., 2024), or bolus location (Pflug et al., 2020), to gain insights into how DBS impacts OD pathophysiology. Additionally, one trial included healthy, age-matched controls (Pflug et al., 2020), enabling clearer attribution of swallowing impairments to PD while accounting for age-related changes in swallowing physiology.

The effect of levodopa on swallowing remains controversial in the literature. Some studies report beneficial effects on swallowing function (Labeit et al., 2020b; Warnecke et al., 2016). For instance, Warnecke et al. (2022) proposed that specific manifestations of oropharyngeal dysphagia are related to dopaminergic deficiency and may therefore improve with levodopa therapy. When findings across the included studies are compared, these same manifestations, such as prolonged oral and pharyngeal transit times and increased pharyngeal residue, also appear to be at least partly responsive to DBS (Ciucci et al., 2008; Kitashima et al., 2013; Lengerer et al., 2012; Cebi et al., 2024). This parallel responsiveness suggests that dopaminergic manifestations of oropharyngeal dysphagia, particularly pharyngeal bradykinesia, may share common underlying pathophysiological mechanisms that can be modulated either through dopamine replacement therapy or through direct basal ganglia deep brain stimulation. Nevertheless, the impact of levodopa on swallowing remains variable, with some studies reporting no clinically meaningful effects (Nascimento et al., 2020) or mixed results across different swallowing parameters (Lim et al., 2008). Discordance between subjective and instrumental swallowing outcomes was observed following DBS. Participant-reported improvement were not always supported by instrumental findings (Kulneff et al., 2013), and conversely, deterioration on instrumental assessments was partly not reflected in subjective reports (Troche et al., 2014). Such discrepancies between subjective and instrumental evaluations have previously been documented in PD (Pflug et al., 2018; Rangwala et al., 2025; Monteiro et al., 2014; Kalf et al., 2012; Nienstedt et al., 2019) and are therefore not unique to the DBS setting. Nevertheless, Yu et al. (2020) suggested a DBS-specific explanation: overall motor improvement after DBS may enhance general patient satisfaction, thereby positively biasing perceptions of swallowing function. Additionally, the choice of statistical analysis for rating scales can affect detection of subtle, yet clinically meaningful, treatment effects. The PAS was the most common measurement tool of swallowing safety across studies, but how the measured values were handled in the statistical analysis varied: some studies used group-level scores only, collapsed categories (e.g., “safe” [PAS 1–2] vs. “unsafe” [PAS 3–8]), or pooled results across consistencies. Essential swallowing protocol details such as consistency types, swallows per consistency, or statistical handling of multiple swallows were often not reported. Such discrepancies can limit statistical power (Borders and Brates, 2020). A recent simulation study (Borders et al., 2025) modeled hypothetical OD intervention trials in PD and demonstrated that using only the worst PAS score or collapsing scores into categories significantly reduces statistical power and introduces bias in effect size estimates. Whereas multilevel models accounting for multiple swallows per participant were more sensitive and accurate in detecting group-level changes over time. These insights are relevant to DBS studies, where mild or variable improvements across stimulation modalities or consistencies may be obscured by summary scoring.

## 4 Future directions

Despite increasing research on the effects of DBS on swallowing function in PD, several important questions remain unanswered. The heterogeneity of study designs, the choice of outcomes and outcome measures, and incomplete reporting of relevant parameters hinder the aggregation of findings. In addition, major gaps in knowledge, including the influence of stimulation settings and anatomical targets, emphasize the need for well-designed, hypothesis-driven studies.

The impact of stimulation settings (frequency, amplitude, pulse width) on swallowing function remains critical but understudied, as most investigations treat these as fixed parameters rather than variables of interest. This may be due to several practical constraints. Systematically varying stimulation parameters increases the study design’s complexity, requiring longer observation periods and larger sample sizes. Ethical considerations may limit experimental manipulation in patients who may risk symptom exacerbation due to parameter changes. Moreover, the multitude of different parameters -encompassing voltage, frequency, pulse width, lead configuration, stimulation directionality- creates a combinatorial challenge difficult to address within a clinical study. Cross-over study designs may enable within-subject comparisons while minimizing risks of prolonged suboptimal stimulation. Additionally, technical limitations such as battery life constraints and programming complexity may impact study designs including frequent parameter adjustments in research protocols. However, the availability of rechargeable pulse generators has alleviated concerns about battery depletion associated with programming exploration (Rizzi et al., 2015). Overcoming these barriers is particularly important for understanding parameter-specific effects on swallowing function. Limited evidence suggests that individual parameters may differentially influence swallowing physiology, potentially enhancing safety and efficiency or causing adverse outcomes. Future research should systematically evaluate how stimulation settings modulate swallowing function and incorporate swallowing outcomes into DBS programming to account for interindividual variability and optimize therapeutic benefit. In terms of outcome measurement, FEES is particularly well suited for real-time optimization of stimulation, with its ability to provide repeated and radiation-free assessments during programming. With recent technological advances, understanding the role of stimulation parameters is essential not only for improving conventional DBS but also for informing the development of adaptive DBS (aDBS). Unlike traditional open-loop DBS, which delivers continuous stimulation irrespective of physiological state, aDBS adjusts stimulation in real time according to dynamic biomarkers such as local field potentials, thereby offering a more individualized approach that may improve both motor and non-motor outcomes. A recent international Delphi expert consensus (Guidetti et al., 2025) emphasized the importance of systematically evaluating the effects of aDBS on non-motor symptoms, including swallowing. However, weak correlations between patient’s subjective perception and objective diagnostic findings complicate symptom-driven aDBS algorithms. Integrating easily detectable real-time biomarkers such as speech-based metrics or acoustic features of swallowing, supported by advances in digital health and wearable sensors (Labeit et al., 2023; Xu et al., 2025), may enable continuous monitoring to enhance aDBS precision for targeting OD. However, stronger correlations with OD must be established before reliable clinical application is possible.

Another important factor in assessing DBS effects on swallowing function is outcome measurement selection. Future studies should address prior limitations, such as solely relying on the swallowing-related items of the UPDRS, which lacks sensitivity for OD. Current OD research is expanding beyond traditional safety-focused scales to also include swallowing efficiency measures and detailed phenotypic/biomechanical characterization (Warnecke et al., 2021). This may have clinical implications, as distinct OD phenotypes are likely to respond differently to specific stimulation settings.

To better understand how DBS parameters affect swallowing, short-term within-subject studies comparing DBS-on versus -off across stimulation settings are needed. Sensitive tools such as the Visual Analysis of Swallowing Efficiency and Safety (VASES) (Curtis et al., 2022) or the FEES-Levodopa-test (Warnecke et al., 2016) could detect setting-dependent differences. Validated outcome measures for classic motor symptoms in the oropharynx (tremor, rigidity, bradykinesia, freezing) are also essential, yet currently remain only provisional or partly lack quantitative scoring. Further refinement and validation of such oropharyngeal motor symptom scores that allow quantification may lead to progress in characterizing DBS but also dopaminergic PD-treatment effects. Since pharyngeal bradykinesia-related OD manifestations partly seem to respond to both levodopa (Labeit et al., 2020b; Warnecke et al., 2022) and DBS, future studies should compare both DBS-off/on and levodopa-off/−on states within the same participants to better characterize these potentially overlapping and synergistic therapeutic mechanisms. Comprehensive insight into parameter effects will support prospective interventional trials with long-term follow-up to capture disease progression, DBS adaptations, and potential remodeling effects of OD. The recently validated DIGEST-FEES tool (Labeit et al., 2024) may be particularly suited for assessing clinically relevant long-term DBS effects on OD in PD.

## 5 Limitations

This review has several limitations. Screening and data extraction were conducted by a single reviewer, no standardized reporting framework was applied, and heterogeneity in study design, outcome measures, electrode placement, and stimulation parameters precluded quantitative synthesis. However, as a narrative review, this work does not aim for systematic synthesis but rather is designed to synthesize emerging and limited evidence.

## 6 Conclusion

OD is a prevalent and relevant complication in PD, yet its role in the context of DBS has received limited attention. Evidence from available studies indicates that DBS may influence swallowing outcomes, but effects vary across stimulation targets, parameters, and OD phenotypes. Importantly, patient-reported outcome measures partly contrast with instrumental measures, underscoring the complexity of assessing treatment effects in this domain.

These discrepancies highlight the need for methodologically rigorous, adequately powered, and hypothesis-driven studies that incorporate standardized instrumental assessments, refined phenotyping of OD, and systematic exploration of stimulation parameters. Future research should consider swallowing outcomes in DBS programming. Moreover, advances in adaptive DBS offer an opportunity to personalize stimulation based on physiological biomarkers, although their integration into swallowing-related outcomes will require validation of robust, sensitive, and clinically meaningful markers.

Although current evidence does not support firm clinical recommendations, swallowing function may warrant consideration during DBS programming in patients with PD. When feasible, instrumental assessment with FEES and the use of sensitive outcome measures (e.g., VASES, DIGEST-FEES, or the FEES-levodopa test) may help identify stimulation-related effects and support individualized clinical decision-making.

In summary, while DBS has clear benefits for motor symptoms in PD, its effects on swallowing remain uncertain. Addressing this gap through carefully designed studies is essential to optimize DBS therapy, improve the management of OD, and ultimately enhance quality of life and safety for patients with PD.
