# Supplementary material for: Effect of deep brain stimulation on dysphagia in Parkinson’s disease: mechanisms, evidence, and outlook
Source: Front Aging Neurosci. 2026 Jan 15;17:1734432. doi: 10.3389/fnagi.2025.1734432 (PMC12852375; doi:10.3389/fnagi.2025.1734432)
Supplement: Supplementary file 1 [file Table_1.docx]

**Supplemental Table 1. Deep brain stimulation electrode coordinates reported in included studies.**

| Study | Target | Point of reference | Side | X (mm) | Y (mm) | Z (mm) |
| --- | --- | --- | --- | --- | --- | --- |
| Cebi et al., 2024 | STN | MCP | left | -7 ≤ x ≤ -12 | -2 ≤ y ≤ − 6 | -6 ≤ z ≤ -10 |
| Cebi et al., 2024 | STN | MCP | right | 7 ≤ x ≤ 12 | -2 ≤ y ≤-6 | -6 ≤ z ≤ -10 |
| Cebi et al., 2024 | STN + SNr | MCP | left | -9.2 ± 1.8 | -5.0 ± 1.1 | -6.5 ± 0.7 |
| Cebi et al., 2024 | STN + SNr | MCP | right | 10.2 ± 1.5 | -4.3 ± 1.3 | -6.5 ± 0.7 |
| Krause et al., 2004 | STN | MCP | Not reported | 10 to 12 | -2 to -3 | -2 to -4 |
| Pflug et al., 2020 | STN + SNr | MCP | left | 10.4 ± 0.9 | 2.8 ± 1.3 | 6.3 ± 1.0 |
| Pflug et al., 2020 | STN + SNr | MCP | right | 10.1 ± 1.7 | 2.7 ± 1.5 | 5.7 ± 1.4 |
| Robertson et al., 2011 | STN | MCP | Not reported | 12 | -4 | -4 |
| Robertson et al., 2011 | GPi | MCP | Not reported | 20 to 21 | 2 | -4 |
| Xu et al., 2018 | STN | MCP | Not reported | 12 | -1 | -4 |
| Zibetti et al., 2007 | STN | MCP | Not reported | 11.6 ± 0.9 | –2.7 ± 0.7 | –3.8 ± 1.1 |
| Troche et al., 2016* | STN | MCP | right | 15.21 | 2.29 | -2.61 |

This table presents the stereotactic coordinates for DBS electrode placements as reported across included studies. Target indicates the anatomical structure (STN = subthalamic nucleus; SNr = substantia nigra; GPi = globus pallidus internus), side specifies laterality and point of reference describes the coordinate system used (MCP = mid-commissural point). Coordinates are presented in the x, y, z format as reported in the original studies.
